# Retraction: ORMDL3 promotes angiogenesis in chronic asthma through the ERK1/2/VEGF/MMP-9 pathway

**DOI:** 10.3389/fped.2025.1640136

**Published:** 2025-06-16

**Authors:** Frontiers Editorial Office

A Retraction of the Original Research Article ORMDL3 promotes angiogenesis in chronic asthma through the ERK1/2/VEGF/MMP-9 pathway By Ding Z, Yu F, Sun Y, Jiao N, Shi L, Wan J and Liu Q (2022). Front. Pediatr. 9:708555. doi: 10.3389/fped.2021.708555

The journal retracts the 16 February 2022 article cited above.

Following publication, concerns were raised regarding the integrity of the images in the published figures. The authors failed to provide a satisfactory explanation during the investigation, which was conducted in accordance with Frontiers' policies. As a result, the data and conclusions of the article have been deemed unreliable and the article has been retracted.

This retraction was approved by the Chief Editors of Frontiers in Pediatrics and the Chief Executive Editor of Frontiers. The authors agree to this retraction.

